# Inhibition of Wnt Signaling Pathways Impairs *Chlamydia trachomatis* Infection in Endometrial Epithelial Cells

**DOI:** 10.3389/fcimb.2017.00501

**Published:** 2017-12-11

**Authors:** Jennifer Kintner, Cheryl G. Moore, Judy D. Whittimore, Megan Butler, Jennifer V. Hall

**Affiliations:** ^1^Department of Biomedical Sciences, Quillen College of Medicine, East Tennessee State University, Johnson City, TN, United States; ^2^Center for Infectious Disease, Inflammation and Immunity, Quillen College of Medicine, East Tennessee State University, Johnson City, TN, United States

**Keywords:** *Chlamydia trachomatis*, co-culture model, Wnt signaling, IWP2, hormones, endometrial epithelial cells, polarized cell culture

## Abstract

*Chlamydia trachomatis* infections represent the predominant cause of bacterial sexually transmitted infections. As an obligate intracellular bacterium, *C. trachomatis* is dependent on the host cell for survival, propagation, and transmission. Thus, factors that affect the host cell, including nutrition, cell cycle, and environmental signals, have the potential to impact chlamydial development. Previous studies have demonstrated that activation of Wnt/β-catenin signaling benefits *C. trachomatis* infections in fallopian tube epithelia. In cervical epithelial cells chlamydiae sequester β-catenin within the inclusion. These data indicate that chlamydiae interact with the Wnt signaling pathway in both the upper and lower female genital tract (FGT). However, hormonal activation of canonical and non-canonical Wnt signaling pathways is an essential component of cyclic remodeling in another prominent area of the FGT, the endometrium. Given this information, we hypothesized that Wnt signaling would impact chlamydial infection in endometrial epithelial cells. To investigate this hypothesis, we analyzed the effect of Wnt inhibition on chlamydial inclusion development and elementary body (EB) production in two endometrial cell lines, Ishikawa (IK) and Hec-1B, in nonpolarized cell culture and in a polarized endometrial epithelial (IK)/stromal (SHT-290) cell co-culture model. Inhibition of Wnt by the small molecule inhibitor (IWP2) significantly decreased inclusion size in IK and IK/SHT-290 cultures (*p* < 0.005) and chlamydial infectivity (*p* ≤ 0.01) in both IK and Hec-1B cells. Confocal and electron microscopy analysis of chlamydial inclusions revealed that Wnt inhibition caused chlamydiae to become aberrant in morphology. EB formation was also impaired in IK, Hec-1B and IK/SHT-290 cultures regardless of whether Wnt inhibition occurred throughout, in the middle (24 hpi) or late (36 hpi) during the development cycle. Overall, these data lead us to conclude that Wnt signaling in the endometrium is a key host pathway for the proper development of *C. trachomatis*.

## Introduction

*Chlamydia trachomatis* genital infections represent the most common notifiable infectious disease in the United States with 1.5 million cases reported in 2015. In fact, *C. trachomatis* has been one of the most prevalent sexually transmitted pathogens in the United States for over two decades (CDC, 2015). Young women, ages 15–29, are disproportionately affected by chlamydial infections, reporting 36% higher rates of infection compared to males of the same age group (CDC, 2015). While many of these infections are clinically mild or asymptomatic, they can contribute to infertility and increase the risk of potentially life-threatening medical emergencies, such as ectopic pregnancy (Peipert, 2003).

*Chlamydia trachomatis* is an obligate intracellular, Gram negative bacterium that possesses a biphasic developmental cycle. Extracellularly, chlamydiae exist in a condensed, infectious form, called elementary bodies (EB). Upon transmission to a new host, EB infect genital luminal and glandular epithelial cells, where they establish a modified endocytic vesicle called an inclusion. After inclusion formation, EB decondense to form larger, metabolically active, non-infectious forms called reticulate bodies (RB). RB undergo several rounds of division within the inclusion before converting into EB, which are released from the infected cell and disseminate to new host cells (Wyrick, 2000; Elwell et al., 2016). Due to their obligate intracellular lifestyle, chlamydiae have evolved mechanisms for interaction with numerous host cell pathways to acquire the essential factors for replication. Interaction of host signaling components with inclusion membrane proteins manipulates host trafficking and signaling so that nutrients are diverted to the RB and prevent host cell death (Elwell et al., 2016). Thus, the general health and signaling environment of genital epithelial cells play a role in chlamydial development.

One group of host pathways that is known to regulate endometrial epithelial cells are the Wnt signaling pathways. At least 20 Wnt signaling ligands have been identified (van der Horst et al., 2012). Wnt signaling plays a major role in embryonic development and regulation of adult tissues (Sonderegger et al., 2010; Wang et al., 2010; van der Horst et al., 2012; Tepekoy et al., 2015). In the endometrium, Wnt signals are secreted from epithelial cells and stromal cells in response to sex hormones (Das et al., 2000; Tulac et al., 2003, 2006). In the canonical pathway, Wnt molecules bind to Frizzled (FZD) receptors on the surface of endometrial cells. Once Wnt is bound to FZD, the receptor interacts with a low density lipoprotein receptor-related protein co-receptor, LRP5/6. This interaction stimulates the recruitment and subsequent disruption of the β-catenin destruction complex, composed of adenomatous polyposis coli (APC), axin, casein kinase Iα and glycogen synthase kinase 3β (GSK-3β). Disruption of the destruction complex by Wnt signaling prevents the degradation of β-catenin that occurs in the absence of Wnt, allowing β-catenin to accumulate in the cytoplasm. β-catenin then translocates to the nucleus where it impacts transcription of genes, including genes that regulate cell survival, migration, and proliferation (Tulac et al., 2003; Wang et al., 2010). Non-canonical Wnt signaling does not elicit action through β-catenin and is less defined due to the multiple signaling pathways that can be activated through this process. The Wnt/planar cell polarity pathway activates GTPases RhoA and Rac, which activate downstream effectors including JNK. The Wnt/Ca^2+^ pathway stimulates the release of calcium from the endoplasmic reticulum. Additionally, non-canonical Wnt signaling can activate ERK and PI3K/AKT signaling (Sonderegger et al., 2010). Thus, cellular signaling via Wnt molecules can impact endometrial cells by altering gene transcription or through downstream activation of additional signaling pathways.

Studies have suggested that Wnt/β-catenin signaling positively influences chlamydial infection by altering epithelial cell structure and/or decreasing host cell apoptosis during infection. Prozialeck et al. demonstrated that N-cadherin/β-catenin cellular junctional complexes were disrupted in *C. trachomatis*-infected cervical epithelial cells and that β-catenin co-localized to the chlamydial inclusion in these cultures (Prozialeck et al., 2002). In fact, knockdown of β-catenin expression in infected host cells decreases both *C. trachomatis* and *C. pneumoniae* infectivity (Kessler et al., 2012; Flores and Zhong, 2015). Activation of Wnt signaling and co-localization of β-catenin to the inclusion has also been demonstrated in *C. trachomatis*-infected ex-planted fallopian tubes. Kessler et al., demonstrated that Wnt activation by chlamydial infection disrupted epithelial cell proliferation, polarity and adhesion in both infected and neighboring epithelial cells, indicating that chlamydial infection impacts uninfected cells via paracrine Wnt signals. (Kessler et al., 2012). Additionally, the *C. pneumoniae* protein Cpn1027 has been shown to interact with the Wnt signaling regulators and members of the β-catenin destruction complex, cytoplasmic activation/proliferation associated protein 2 (caprin 2) and GSK-3β, potentially increasing β-catenin accumulation in the infected cell and expression of β-catenin-regulated anti-apoptotic genes (Flores and Zhong, 2015).

A unique feature of epithelial cells lining the endometrium of the female genital tract (FGT) is that they are stimulated to proliferate, differentiate, and are shed in response to hormone-induced signaling events during the menstrual cycle. Interestingly, modulation of Wnt signaling pathways by sex hormones plays a role in this cyclic remodeling of the endometrium. (Sonderegger et al., 2010; Wang et al., 2010; van der Horst et al., 2012; Tepekoy et al., 2015). Given the previous observations that Wnt/β-catenin signaling impacts chlamydial infection in fallopian tubes and in cervical cells, we sought to determine if inhibiting Wnt signals in endometrial epithelial cells has a negative influence on chlamydial development.

## Methods

### Culture of endometrial epithelial cell lines and *Chlamydia*

Ishikawa (IK), Hec-1B and an endometrial stromal cell line (SHT-290) were maintained as described previously (Hall et al., 2011). Briefly, IK, Hec-1B, and SHT-290 cells were maintained in phenol-red free Dulbecco's modified Eagle's medium (DMEM; Gibco) with 1x glutamax (Gibco). IK and Hec-1B medium was supplemented with 5 or 10% FBS, respectively. SHT-290 medium was supplemented with 2% charcoal-stripped FBS (CSFBS). *C. trachomatis* Serovar E stocks were prepared in Hec-1B cells grown in bead culture as previously described (Guseva et al., 2007). Short tandem repeat profiling was performed by the American Type Culture Collection to authenticate the identity and/or origin of all cell lines used in this study (data not shown). All cell lines were tested for *Mycoplasma* by PCR and found to be free of contamination (data not shown).

### Wnt inhibitor exposure

Ishikawa (IK) cells were cultured on Matrigel-coated filters (Corning) until they reached 75% confluency. The IK cultures were placed into wells containing SHT-290 monolayers growing on the bottom of the well so that the IK and SHT-290 monolayers were in close proximity to each other, but not touching. Co-cultures were replenished with SHT-290 medium containing DMSO or 5 uM IWP2 (Tocris) and incubated at 37°C for 48 h. At this time, the medium was removed from the cultures and an inoculum of *C. trachomatis* EB in 2SPG (0.2 M sucrose, 0.02 M phosphate buffer, and 5 mM l-glutamine) was overlaid on the IK monolayers. A low MOI of 0.03 IFU/cell was used for all studies so that the percentage of infected cells could be accurately determined. SHT-290 monolayers were overlaid with an equal volume of mock inoculum (2SPG) to prevent drying. The cultures were incubated for 1 h at 35°C before the inoculum was removed. The cultures were refed with the appropriate experimental medium containing DMSO or IWP2 and incubated for 48 h at 35°C before collection for various analyses. Alternatively, replicate Hec-1B and/or IK nonpolarized monolayers were exposed to IWP2 or 25 uM IWR-1 (Selleckchem) as described above or at 0, 24, or 36 h post *Chlamydia* infection (hpi). The chlamydia-infected, DMSO, IWP2 or IWR-1-exposed Hec-1B and/or IK monolayers were collected at 44 hpi. In duplicate experiments, samples were washed and refed with culture medium containing neither DMSO nor IWP2 at 44 hpi. These cultures were incubated for an additional 72 h at 35°C before collection. Inhibitor concentrations were chosen based upon the manufacture's and published literature in combination with preliminary dose response curves in which the viability of host cells was determined by monitoring cell growth and Live/Dead assay (Kessler et al., 2012; Lee et al., 2015).

### Percent infectivity

Infected IK or Hec-1B samples were fixed with methanol and stained for chlamydial inclusions using Pathfinder anti-chlamydia MOMP stain (BioRad). Cell nuclei were stained with DAPI (ThermoFisher). Stained coverslips and filters were mounted on glass slides. Chlamydial inclusions and nuclei were visualized on a Ziess Axiovert Discovery Microscope at 400x magnification. In each experiment, the number of inclusions and nuclei were counted in 15 random fields from triplicate samples and the average percentage of infected cells was calculated per condition.

### EB titer analysis

*Chlamydia trachomatis*-infected monolayers were scraped into the culture medium and frozen at −80°C. EB released from the cells by freeze/thaw and sonication were diluted in culture medium and used to infect HeLa 229 monolayers grown on coverslips by spin infection (1 h, 1,100x g). Triplicate infected monolayers were incubated for 48 h at 35°C before methanol fixation and staining with Pathfinder anti-chlamydia MOMP stain (BioRad). Total inclusions were counted on each coverslip using a Ziess Axiovert Discovery Microscope at 200x magnification. The average number of inclusion forming units (IFU)/ml was calculated per sample and experimental condition.

### Inclusion size measurement

Triplicate chlamydia-infected IK and Hec-1B nonpolarized monolayers grown on coverslips or polarized IK monolayers from IK/SHT-290 co-cultures were stained with Pathfinder anti-chlamydia MOMP stain (BioRad). Five images of random fields were taken of each sample at 630x magnification using a Ziess Axiovert Discovery Microscope. The area of 10–12 inclusions/sample was measured using Zen Professional Imaging Software (Ziess). The average inclusion size was determined for each experimental condition.

### Live/dead assay

Ishikawa (IK) cultures were exposed to DMSO or IWP2 for a period of 1–4 days at 35°C. Cell death in the cultures was assessed using the Live/Dead Reduced Biohazard Viability/Cytotoxicity Kit (Molecular Probes) according to the manufacturer's instructions.

### Confocal microscopy analysis

Infected IK monolayers from IK/SHT-290 co-cultures exposed to DMSO or IWP2 were stained for percent infectivity analysis as described above. Alternatively, IK and Hec-1B nonpolarized monolayers were stained with anti-β-catenin (Cell Signaling), and anti-chlamydial MOMP (Meridian LifeSciences) primary antibodies followed by staining with rabbit-anti-mouse Alexa Fluor 488 (Molecular Probes), rabbit-anti-goat Alexa Fluor 594 (Molecular Probes) secondary antibodies, and DAPI (ThermoFisher) stain. Confocal images of chlamydial inclusions were captured on a Leica TCS SP8 with Leica LASX software at 630x or 1,000x magnification. Imaris imaging and Leica LASX software were used to analyze the images. All images used in intensity measurements were taken at a standard exposure. Digital slices representing the center depth of 24 inclusions/condition were used to determine the intensity of β-catenin staining inside the inclusion. The intensity of β-catenin staining within a region of the host cell equivalent to the area of each inclusion was determined to assess the ratio of β-catenin staining inside the inclusion compared to the cell cytoplasm and/or nucleus.

### Transmission electron microscopy

Infected IK monolayers from IK/SHT-290 co-cultures exposed to DMSO or IWP2 were fixed with glutaraldehyde in 0.1 M Cacodylate buffer (EM Sciences) for 24 h. The monolayers were then collected, agar enrobed, and stained with osmium tetroxide and uranyl acetate before alcohol and propylene oxide dehydration. Dehydrated samples were embedded in EPON (Polysciences, Inc Araldite 502/PolyBed 812 Kit) embedding medium (Wyrick et al., 1994). Thin sections were examined on a Tecnai 10 (FEI) transmission electron microscope operating at 60–80 kV.

### Western blot analysis

Infected IK and SHT-290 monolayers from IK/SHT-290 co-cultures were collected in 8 M urea. The protein samples were examined by SDS-PAGE electrophoresis for Sypro Ruby (BioRad) staining and Western Blot analysis as previously described (Deka et al., 2006). Blots were probed with anti-β-catenin antibodies (Cell Signaling). Protein accumulation in each experiment was quantified from triplicate samples using a BioRad G-box and SynGene software. β-catenin accumulation was normalized to the total amount of protein detected in each sample by Sypro Ruby staining.

### Statistical analysis

All experiments contained three biological replicates and were independently repeated a minimum of three times. Values from independent experiments were averaged and presented as the mean ± the standard error of the mean (SEM). Means were compared by Analysis of Variance (ANOVA) and independent two-sample *T*-tests using MiniTab Version 17 Statistical Software and Microsoft Excel. Comparisons with *p* ≤ 0.05 were considered significantly different.

## Results

### Inhibition of Wnt in IK/SHT-290 co-culture decreases chlamydial infectivity and EB production

**Figure 1:**
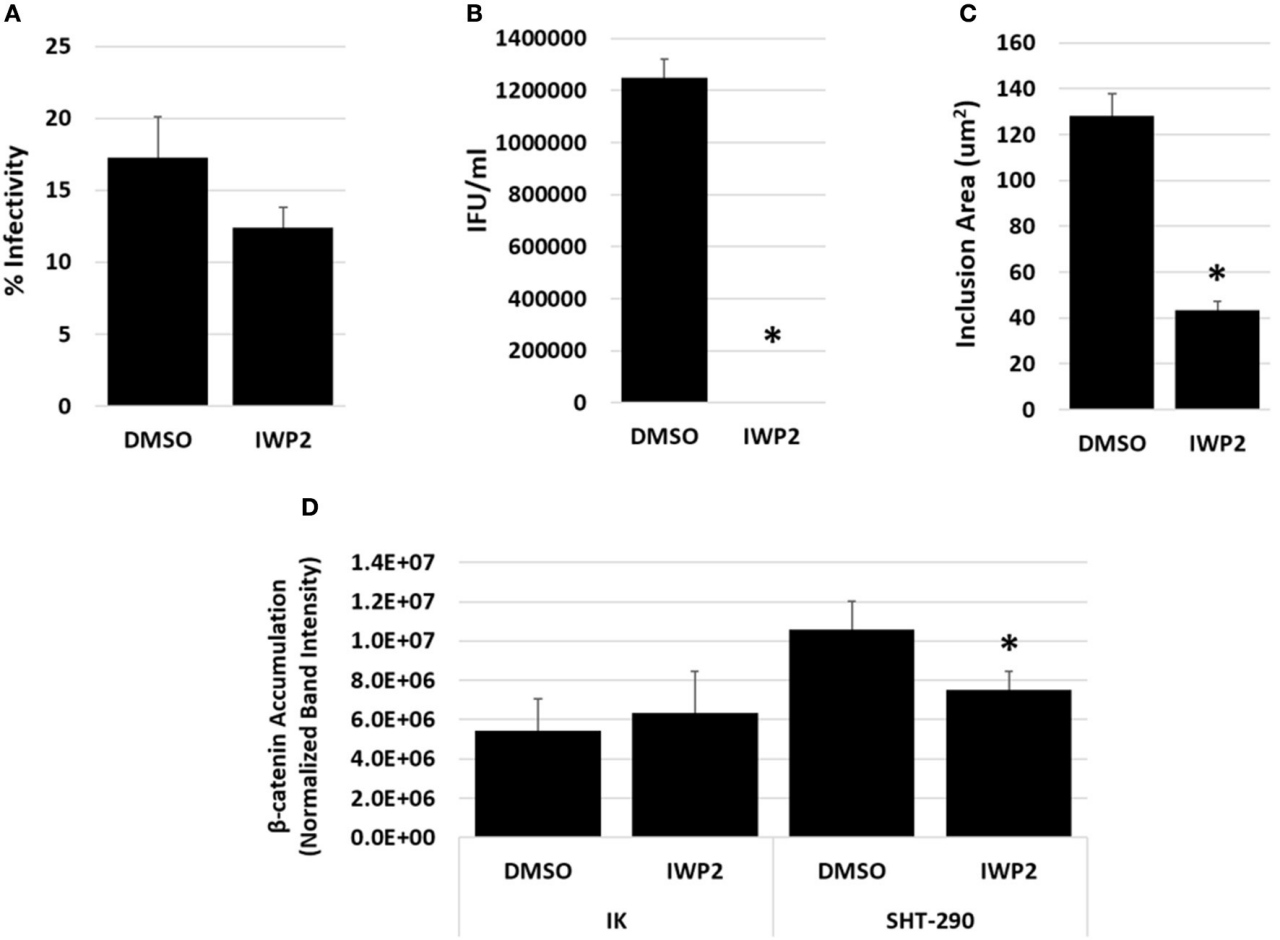
Impact of Wnt inhibition on chlamydial infection in IK/SHT-290 co-culture. Replicate IK/SHT-290 co-cultures were exposed to DMSO or the Wnt inhibitor, IWP2, prior to and following *C. trachomatis* infection. At 44 hpi, the infected IK monolayers and SHT-290 monolayers were collected and processed for **(A)** percent infectivity analysis, **(B)** EB titer assay, **(C)** inclusion size measurement, **(D)** and Western blot analysis of β-catenin accumulation. The data shown represent three independent experiments with 3 biological replicates per experiment. Comparison of means was performed using a 2-sample *T*-test for independent samples. *P* ≤ 0.05 were considered statistically significant. ^*^Indicates a significant difference (*p* ≤ 0.05) from the DMSO control.

To determine if Wnt signaling is important for chlamydial development in the endometrium, we examined the effects of a Wnt inhibitor, IWP2 on chlamydial infection in an endometrial epithelial cell (IK)/stromal (SHT-290) cell co-culture model. This physiologically relevant co-culture model incorporates paracrine regulatory signaling events that occur between endometrial stromal and epithelial cells (Arnold et al., 2001, 2002). IWP2 is a small molecule inhibitor of Wnt protein production that has the ability to inhibit both the canonical, Wnt/β-catenin signaling pathways and non-canonical Wnt signaling pathways (Chen et al., 2009). Replicate IK/SHT-290 co-cultures were prepared as described in the methods. The co-cultures were exposed to DMSO or 5 uM IWP2 48 h prior to and following *C. trachomatis* infection. Replicate samples were harvested at 44 hpi for chlamydial infectivity, EB titer analysis, inclusion size measurements and Western blot analysis of β-catenin accumulation. In a separate study, Live/Dead assays revealed that prolonged exposure to 5 uM IWP2 did not increase IK cell death significantly compared to the DMSO control (data not shown). Chlamydial infectivity was lowered in the presence of Wnt inhibition compared to DMSO exposure, although not to a significant degree (Figure 1A, *p* = 0.20). Inhibition of Wnt by IWP2 significantly decreased the production of infectious EB during IK/SHT-290 co-culture (Figure 1B, *p* = 0.002). Inclusion size was also significantly decreased in cultures exposed to IWP2 compared to DMSO (Figure 1C, *p* = 0.0002). Accumulation of β-catenin was significantly decreased in SHT-290 cells (*p* = 0.04), but not in IK cells (*p* = 0.60) from infected IK/SHT-290 co-cultures (Figure 1D, Supplemental Figure 1).

**Figure 2:**
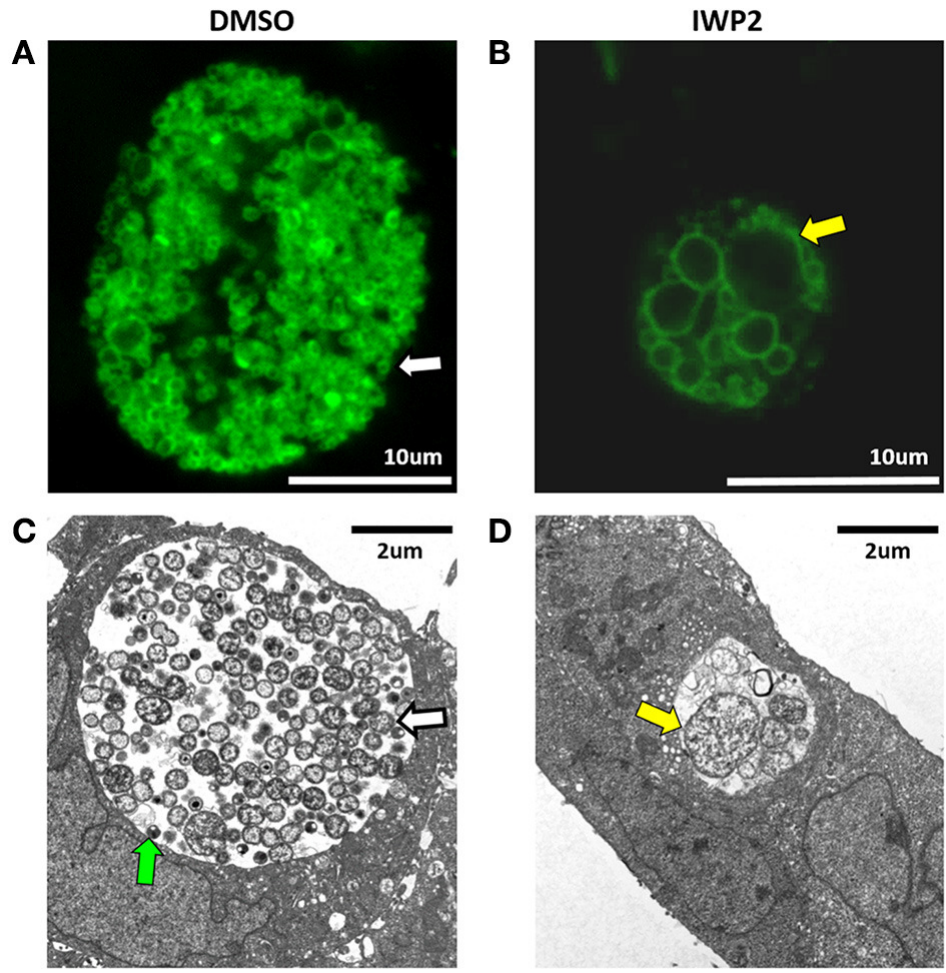
Confocal and transmission electron microscopy of chlamydia-infected IK cells cultivated in the presence of DMSO or IWP2 during IK/SHT-290 co-culture. Replicate IK/SHT-290 co-cultures were exposed to DMSO or the Wnt inhibitor, IWP2, prior to and following *C. trachomatis* infection. At 44 hpi, the infected IK monolayers were fixed, stained for chlamydia and visualized by confocal microscopy (**A,B**, green) or processed for TEM **(C,D)**. Shown are representative images of chlamydial inclusions in infected IK cells exposed to DMSO **(A,C)** or IWP2 **(B,D)**. Confocal images were captured at 1,000x magnification. TEM images were captured at 7,000x magnification. EB, normal RB, and aberrant RB are indicated by the green, white and yellow arrows, respectively.

Examination of chlamydia-infected IK monolayers exposed to DMSO or IWP2 during co-culture by TEM and confocal microscopy reveal that Wnt inhibition caused inclusions to be smaller in size and contain aberrant RB (Figures 2B,D, yellow arrows) compared to DMSO-exposed controls, which had large inclusions, containing numerous RB (white arrows) and EB (green arrow) (Figures 2A,C). These data indicate that inhibition of Wnt signaling in IK/SHT-290 co-cultures inhibits chlamydial development and production of infectious progeny. Moreover, formation of aberrant RB suggests that Wnt inhibition may cause developing chlamydiae to enter into a stressed or persistent state. Given that β-catenin accumulation was decreased in SHT-290 cells but not in IK cells, these data suggest that Wnt inhibition during co-culture impacts chlamydial infection by inhibiting paracrine stromal cell effectors released in response to canonical Wnt/β-catenin activation. Alternatively, inhibition of non-canonical Wnt signaling pathways or alterations in β-catenin localization in infected IK cells may contribute to the negative effect of Wnt inhibition on chlamydial infection.

### Inhibition of Wnt in nonpolarized IK and Hec-1B cells decreases infectivity and EB progeny production

**Figure 3:**
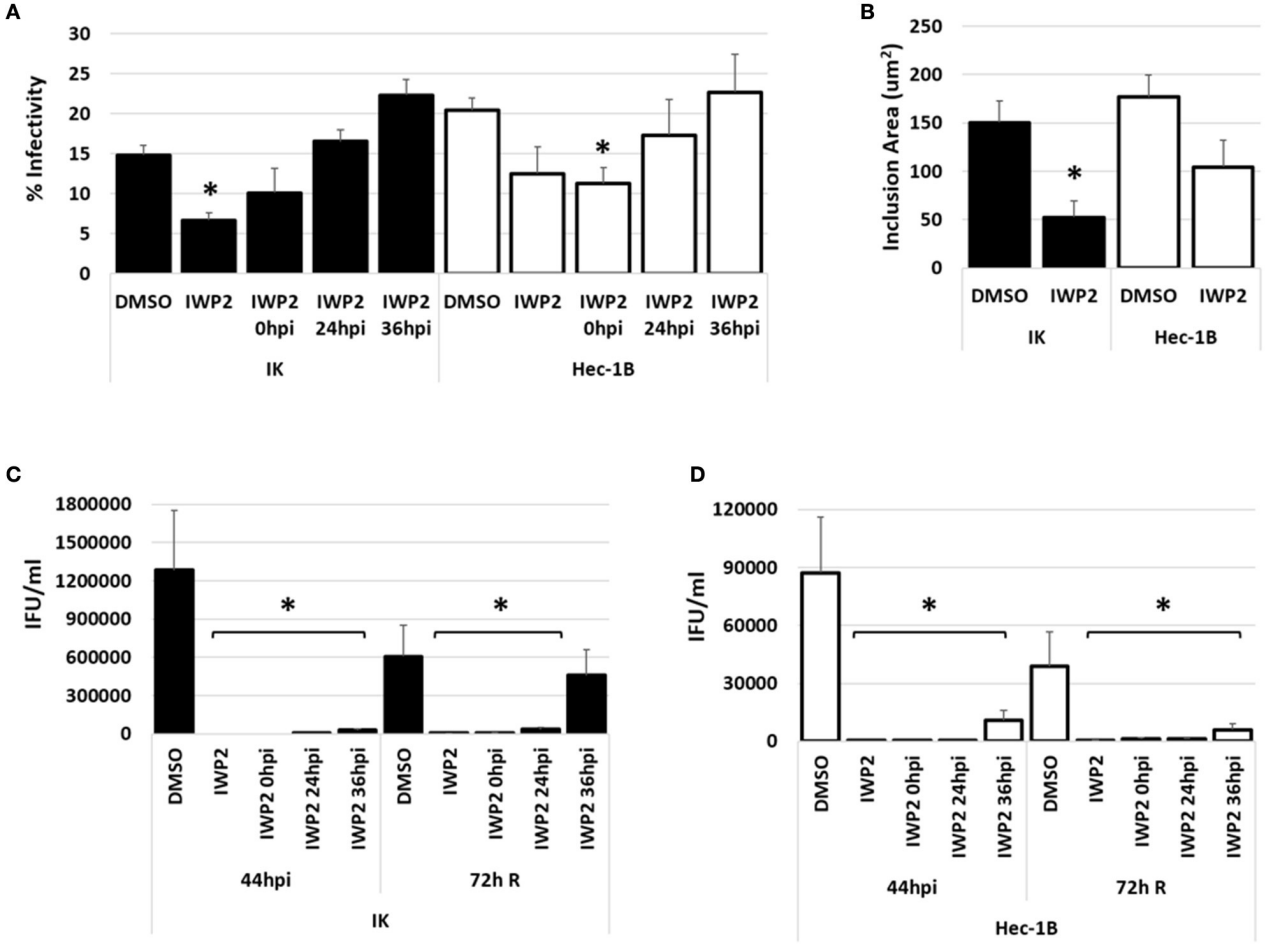
Impact of Wnt inhibition by IWP2 on chlamydial infection in nonpolarized endometrial epithelial cells, IK and Hec-1B. Nonpolarized IK or Hec-1B cell monolayers were exposed to DMSO or IWP2 prior to and/or after (0, 24, or 36 hpi) *C. trachomatis* infection. Samples were harvested for infectivity and EB titer assay at 44 hpi. Replicate samples were replenished with fresh medium at 44 hpi and collected 72h after removal of DMSO or IWP2 from the cultures (72 h R). **(A)** Chlamydial percent infectivity and **(B)** average inclusion size in IK (black bars) or Hec-1B (white bars) cultures at 44 hpi. **(C,D)** EB progeny production from infected IK **(C)** or Hec-1B **(D)** cultures exposed to DMSO or IWP2 at 44 hpi and 72 h R. Comparison of means was performed using ANOVA and 2-sample *T*-tests for independent samples. ^*^Indicates a significant difference (*p* ≤ 0.05) from the DMSO control.

To ensure that our observed results were not limited to IWP2-exposed, chlamydia-infected IK/SHT-290 co-cultures, we repeated our studies using nonpolarized IK cells and a second endometrial cell line, Hec-1B. Nonpolarized IK or Hec-1B cell monolayers were exposed to DMSO or IWP2 48h prior to *C. trachomatis* infection. Replicate cultures were exposed to IWP2 at 0, 24, or 36 h post *C. trachomatis* infection. Samples were harvested for various analyses at 44 hpi. Data from chlamydial persistence models demonstrate that aberrant chlamydiae recover infectivity from this stressed state when the persistence inducer is removed (Hogan et al., 2004; Vanover et al., 2010). Given that we observed aberrant RB morphology during IWP2 exposure in IK/SHT-290 co-culture, we sought to determine if *C. trachomatis* could recover from Wnt inhibition by IWP2. Duplicate samples were washed with sterile phosphate buffered saline and refed with medium containing no diluent or inhibitor. These samples were allowed to recover for 72 h (72 h R) before collection for EB titer analysis. Compared to DMSO-exposed controls, chlamydial infectivity was significantly decreased in IK cultures when exposed to IWP2 before infection (*p* < 0.001). However, addition of the Wnt inhibitor at times after infection did not significantly impact chlamydial inclusion development in IK cells (Figure 3A, black bars). In Hec-1B cultures, inclusion development was decreased compared to the control when IWP2 was added before or at 0 hpi (*p* = 0.013) chlamydial infection; however, Wnt inhibition had no significant effect on continued inclusion development when IWP2 was added to Hec-1B cultures at 24 or 36 hpi (Figure 3A, white bars). Inhibition of Wnt significantly decreased the average size of inclusions in IK cells compared to DMSO exposure (Figure 3B, *p* = 0.0046). Inclusion size in Hec-1B cells was also decreased in response to Wnt inhibition, but not to a significant degree (Figure 3B, *p* = 0.07). EB production was suppressed in both IK (Figure 3C) and Hec-1B (Figure 3D) cultures exposed to IWP2 at 44 hpi (*p* < 0.0001). Inclusion size in IWP2-exposed samples was increased at 72 h R compared to 44 hpi (Supplemental Figure 2), but EB production did not recover appreciably in IK or Hec-1B cells following the removal of the Wnt inhibitor except in IK cultures exposed to IWP2 at 36 hpi (Figures 3C,D). Western blot analysis demonstrated that IWP2 exposure did not significantly alter accumulation of β-catenin in nonpolarized IK or Hec-1B cultures at 44 hpi (Supplemental Figure 3).

**Figure 4:**
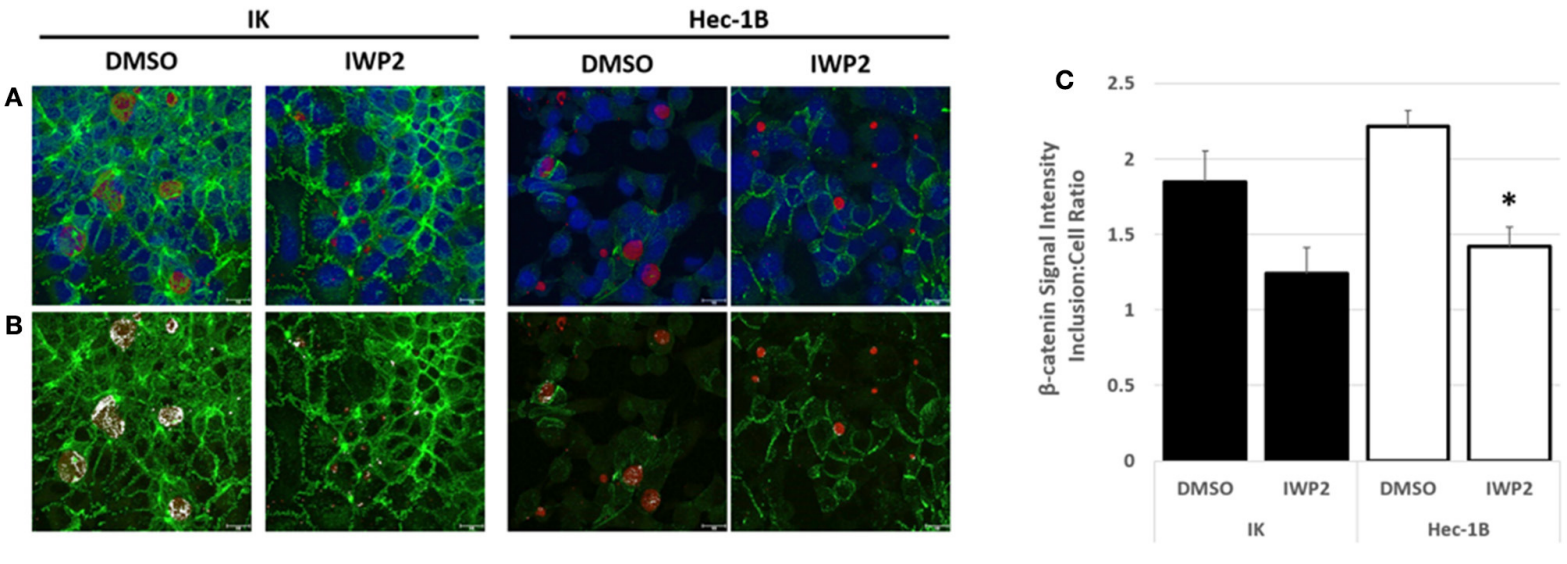
Confocal microscopy of chlamydia-infected IK and Hec-1B monolayers cultivated in the presence of DMSO or IWP2. Nonpolarized IK and Hec-1B cell monolayers were exposed to DMSO or IWP2 and infected with *C. trachomatis*. At 44 hpi the cultures were methanol fixed and subjected to immunofluorescent staining. Confocal microscopy images were captured at 630x magnification. **(A)** Representative images depicting fluorescently labeled β-catenin (green), chlamydial MOMP (red), and host cell nuclei (DAPI, blue). **(B)** Duplicates of the representative images in panel A are shown. Areas in which β-catenin and MOMP co-localized are indicated in white. **(C)** The average ratio of β-catenin staining intensity ±SEM measured in the center of an inclusion compared to an equal area of the host cell. ^*^Indicate a significant difference (*p* ≤ 0.05) between the β-catenin staining intensity inclusion:cell ratio detected in DMSO verses IWP2-exposed samples. Black and white bars represent IK and Hec-1B samples respectively.

Confocal microscopy examination of fluorescently labeled β-catenin in DMSO or IWP2-exposed, chlamydia-infected IK or Hec-1B cultures revealed that β-catenin localized to chlamydial inclusions under both experimental conditions (Figures 4A,B). Although elevated by approximately 2 fold, the intensity of β-catenin staining observed within inclusions was not significantly different than that observed in the host cell in the DMSO-exposed controls, suggesting that while β-catenin is present in the inclusion it was not concentrated there (Figure 4C). IWP2 exposure decreased β-catenin localization in the inclusion compared to the DMSO controls in infected Hec-1B cultures (Figure 4C, white bars, *p* = 0.003). Overall, these data indicate that inhibition of Wnt signaling in nonpolarized endometrial epithelial cells severely inhibits chlamydial inclusion development and infectious progeny production. Importantly, the detrimental effects of Wnt inhibition on *C. trachomatis* was evident no matter if the inhibition occurred before infection, or during early, mid, or late infection, indicating that Wnt signaling is important at every stage of the chlamydial intracellular development cycle.

### Inhibition of Wnt in nonpolarized IK cells by IWR-1 decreases infectivity and EB progeny production

**Figure 5:**
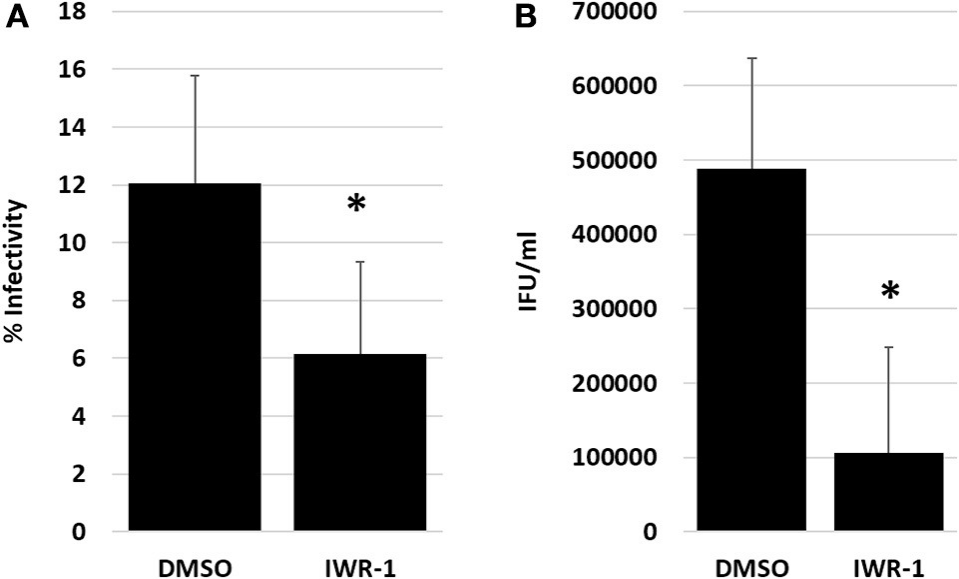
Impact of Wnt inhibition by IWR-1 on chlamydial infection in nonpolarized IK cells. Nonpolarized IK monolayers were exposed to DMSO or IWR-1 at 0 h post *C. trachomatis* infection. Samples were harvested for infectivity and EB titer assay at 44 hpi. **(A)** Chlamydial percent infectivity. **(B)** EB progeny production from infected IK. Comparison of means was performed 2-sample *T*-tests for independent samples. ^*^Indicates a significant difference (*p* ≤ 0.05) from the DMSO control.

To confirm that the observed effects of Wnt inhibition on chlamydial infection were not limited to IWP2 exposure, we examined the effect of another Wnt inhibitor, IWR-1, on chlamydial infection in endometrial epithelial cells. IWR-1 is a small molecule that inhibits canonical Wnt activity via stabilization of the β-catenin destruction complexes (Chen et al., 2009). Nonpolarized IK cell monolayers were exposed to DMSO or IWR-1 at 0 h post *C. trachomatis* infection. Samples were harvested for analysis of infectivity and EB production at 44 hpi. As shown in Figures 5A,B, IWR-1 exposure significantly decreased *C. trachomatis* percent infectivity (*p* = 0.01) and the production of infectious EB (*p* = 0.001) from the cultures. These data confirm the importance of Wnt/β-catenin signaling on chlamydial infection in endometrial cells.

## Discussion

Here, we have demonstrated that Wnt signaling is an essential pathway for chlamydial development in endometrial epithelial cells. Inhibition of Wnt significantly decreases chlamydial inclusion development, causing decreased percent infectivity and inclusion size. Furthermore, RB that formed during Wnt inhibition were aberrant in morphology. Inhibition of Wnt also caused a dramatic decrease in EB formation regardless of the time that Wnt was inhibited during the developmental cycle. Recovery of progeny formation following the removal of the Wnt inhibitor was only observed when the Wnt inhibitor was added late during chlamydial development. These data indicate that *C. trachomatis* may enter a persistent state or be killed when Wnt signals are inhibited in the host cell. Overall these data lead us to conclude that Wnt signaling is a key host pathway for the proper development of *C. trachomatis* in the endometrium. Our observations support previous work that indicate activation of Wnt signaling and/or β-catenin is important for chlamydial development in other areas of the FGT, including the fallopian tubes and cervical cells (Prozialeck et al., 2002; Kessler et al., 2012). Conversely, Kessler et al., found that IWP2 exposure increased chlamydial Hsp60 accumulation in infected fallopian tube tissue. The authors concluded that this result indicated increased chlamydial replication during Wnt inhibition (Kessler et al., 2012). However, continued or increased accumulation of Hsp60 has been observed in several models of chlamydial persistence or stress, including interferon-γ, viral co-infection, iron limitation, and monocyte infection (Beatty et al., 1993, 1994; Raulston, 1997; Gerard et al., 2004; Deka et al., 2006). While certainly not the only possibility, it is intriguing to speculate that IWP2 caused chlamydiae to enter a stressed state in fallopian tissues rather than increase chlamydial replication. Our observation that RB become aberrant during IWP2 exposure in endometrial cells supports this alternative conclusion. Together, data from the current study and previous studies highlight an important role for Wnt signaling in *C. trachomatis* infections throughout the entire tract.

Previous research has demonstrated that female sex hormones, estrogen and progesterone, can influence chlamydial development (Rank et al., 1982, 1993; Bose and Goswami, 1986; Sweet et al., 1986; Maslow et al., 1988; Wyrick et al., 1993; Guseva et al., 2003, 2007; Hall et al., 2011; Kintner et al., 2015). Using the IK/SHT-290 co-culture model of *C. trachomatis* infection, we have demonstrated that estrogen increases chlamydial development whereas progesterone negatively impacts *C. trachomatis* infection by unknown molecular mechanisms. These studies revealed the paracrine stromal cell signaling molecules released in response to hormones play a role in hormone-induced effects on chlamydial development (Hall et al., 2011; Kintner et al., 2015). Wnt signaling pathways are essential to the cyclic replenishment of the endometrium during the menstrual cycle. Following loss of the functional epithelial layer of the endometrium during menses, estrogen stimulates proliferation of epithelial cells in the basal endometrial layer. During the proliferative phase, estrogen stimulates the transcription of a variety of genes and activates cellular signaling pathways to induce cellular division and migration (Sonderegger et al., 2010; Wang et al., 2010; van der Horst et al., 2012; Tepekoy et al., 2015). Activation of both canonical and non-canonical Wnt signaling by estrogen is thought to play a central role in estrogen's proliferative and regulatory effects on endometrial epithelia (Das et al., 2000; Gunin et al., 2004; Hou et al., 2004; Wang et al., 2009; Sonderegger et al., 2010). Following the estrogen-dominant phase of the menstrual cycle, progesterone is produced during the secretory phase. Progesterone counterbalances the proliferative effects of estrogen on the endometrium to promote cellular differentiation in preparation for embryo implantation (Sonderegger et al., 2010). One mechanism employed by progesterone to inhibit the effects of estrogen on endometrial epithelial cell growth and proliferation is increasing gene expression of Wnt signaling inhibitors, dickkopf homolog 1 (DKK1), forkhead box O1 (FOXO1), and miR-152 (Wang et al., 2009; Nie et al., 2017). Thus, growth and differentiation of endometrial epithelium is directly linked to hormone-induced regulation of Wnt signaling networks.

Given that Wnt signaling is important to chlamydial development in the endometrium and that hormones naturally regulate Wnt signaling as a part of the menstrual cycle, it is intriguing to hypothesize that hormonal regulation of Wnt signaling may contribute to the impact of estrogen and progesterone on *C. trachomatis* infection. The demonstrated effects of Wnt/β-catenin signaling on chlamydial development could be attributed to hormonal modulation of Wnt signaling. For example, Kessler et al. proposed that *Chlamydia*-activated Wnt/β-catenin signaling in fallopian tube epithelia altered host cell adhesion and polarity (Kessler et al., 2012). Prozialeck et al. also demonstrated that sequestration of β-catenin to the inclusion altered host cell junctional complexes (Prozialeck et al., 2002). Estrogen signaling in the endometrium also contributes to endometrial epithelial cell junction remodeling during cellular proliferation and migration (Tulac et al., 2003; van der Horst et al., 2012). Future studies investigating the regulation of Wnt signaling pathways by steroid hormones in *C. trachomatis*-infected endometrial epithelial cells will further illuminate the importance of this intricate and complex system to chlamydial growth and pathogenesis.

## Author contributions

JK, CM, JW, MB, and JH contributed substantially to the design, execution, and/or data collection/analysis of the experiments within this study. JH drafted the manuscript. All authors contributed to revision and final approval of the manuscript.

### Conflict of interest statement

The authors declare that the research was conducted in the absence of any commercial or financial relationships that could be construed as a potential conflict of interest.

## Supplementary material

Supplemental Figure 1 Images of representative β-catenin Western blots from IK/SHT-290 co-cultures.

Supplemental Figure 2 Example images of chlamydial inclusions at 44 hpi and 72 h R from DMSO or IWP2-exposed IK and Hec-1B cultures. Chlamydial inclusions (green) were stained with BioRad anti-MOMP Pathfinder stain. Cell nuclei (blue) were stained with DAPI. The images were captured at 400x magnification.

Supplemental Figure 3 **(A)** Images of representative β-catenin Western blots from chlamydia-infected, DMSO or IWP2-exposed, IK and Hec-1B cultures at 44 hpi. **(B)** Western blot analysis of β-catenin accumulation. β-catenin accumulation was normalized to total protein detected by Sypro Ruby staining as described in the methods. Comparison of means was performed using a 2-sample *T*-test for independent samples. There was no statistical difference in β-catenin accumulation between DMSO and IWP2-exposed, *C. trachomatis*-infected IK (*p* = 0.38) or Hec-1B (*p* = 0.92) cultures.
