# Supplementary figures and images for: Inhibition of Wnt Signaling Pathways Impairs Chlamydia trachomatis Infection in Endometrial Epithelial Cells
Source: Front Cell Infect Microbiol. 2017 Dec 11;7:501. doi: 10.3389/fcimb.2017.00501 (PMC5732136; doi:10.3389/fcimb.2017.00501)

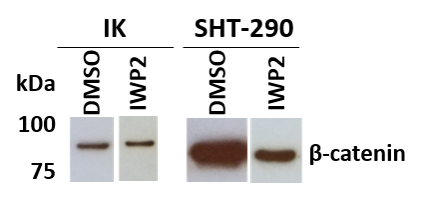

Supplement: Supplemental Figure 1 — Images of representative β-catenin Western blots from IK/SHT-290 co-cultures. [file Image1.TIF]

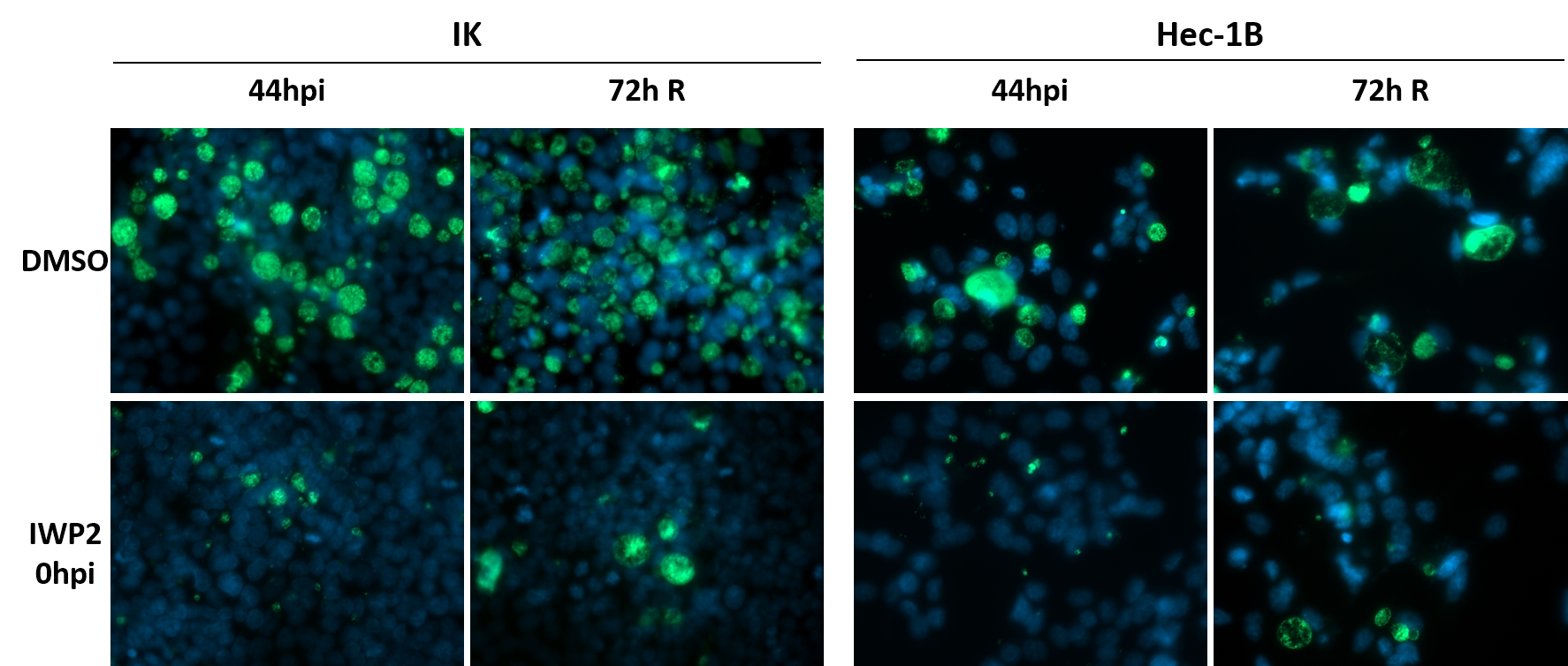

Supplement: Supplemental Figure 2 — Example images of chlamydial inclusions at 44 hpi and 72 h R from DMSO or IWP2-exposed IK and Hec-1B cultures. Chlamydial inclusions (green) were stained with BioRad anti-MOMP Pathfinder stain. Cell nuclei (blue) were stained with DAPI. The images were captured at 400x magnification. [file Image2.TIF]

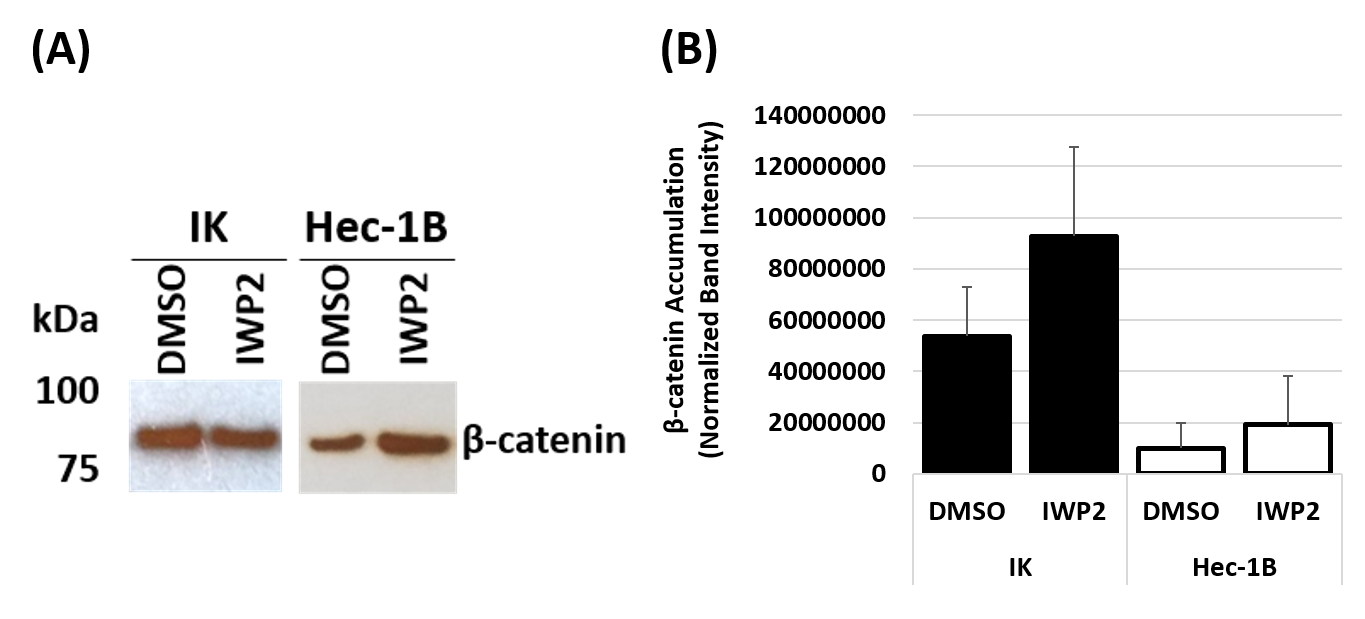

Supplement: Supplemental Figure 3 — (A) Images of representative β-catenin Western blots from chlamydia-infected, DMSO or IWP2-exposed, IK and Hec-1B cultures at 44 hpi. (B) Western blot analysis of β-catenin accumulation. β-catenin accumulation was normalized to total protein detected by Sypro Ruby staining as described in the methods. Comparison of means was performed using a 2-sample T-test for independent samples. There was no statistical difference in β-catenin accumulation between DMSO and IWP2-exposed, C. trachomatis-infected IK (p = 0.38) or Hec-1B (p = 0.92) cultures. [file Image3.TIF]
